# Osmophores and floral fragrance in *Anacardium humile* and *Mangifera indica* (Anacardiaceae): an overlooked secretory structure in Sapindales

**DOI:** 10.1093/aobpla/ply062

**Published:** 2018-10-05

**Authors:** Elisabeth Dantas Tölke, Julien B Bachelier, Elimar Alves de Lima, Marcelo José Pena Ferreira, Diego Demarco, Sandra Maria Carmello-Guerreiro

**Affiliations:** 1 Departamento de Biologia Vegetal, Instituto de Biologia, Universidade Estadual de Campinas – UNICAMP, CEP Campinas, São Paulo, Brazil; 2 Institute of Biology, Structural and Functional Plant Diversity Group, Freie Universität Berlin, Altensteinstrasse, Berlin, Germany; 3 Departamento de Botânica, Instituto de Biociências, Universidade de São Paulo, CEP 05508-090 São Paulo, São Paulo, Brazil

**Keywords:** Cashew family, floral scent, osmophores, pollinator attraction, secretory structures

## Abstract

Flowers of Anacardiaceae and other Sapindales typically produce nectar, but scent, often associated with a reward for pollinators, has surprisingly been mentioned only rarely for members of the family and order. However, flowers of *Anacardium humile* and *Mangifera indica* produce a strong sweet scent. The origin and composition of these floral scents is the subject of this study. Screening of potential osmophores on the petals and investigations of their anatomy were carried out by light, scanning and transmission electron microscopy. The composition of the floral fragrance was characterized by gas chromatography–mass spectrometry. In both species, the base of the adaxial side of each petal revealed specialized secretory epidermal cells which are essentially similar in structure and distinct from all other neighbouring cells. These cells also showed evidence of granulocrine secretory mechanisms and slight specific variations in their subcellular apparatus coinciding with the respective composition of the floral fragrance, predominantly composed of sesquiterpenes in *A. humile* and monoterpenes in *M. indica*. This study reports the presence of osmophores for the first time in flowers of Anacardiaceae and confirms the link between the ultrastructural features of their secretory cells and the volatiles produced by the flowers. The flowers of most Sapindales, including Anacardiaceae, are nectariferous. However, the presence of osmophores has only been described for very few genera of Rutaceae and Sapindaceae. Both the occurrence of osmophores and fragrance may have largely been overlooked in Anacardiaceae and Sapindales until now. Further studies are needed to better understand the nature and diversity of the interactions of their nectariferous flowers with their pollinators.

## Teaser

This study focuses on the first report of osmophores in flowers of the economically important family Anacardiaceae. Flowers of *Anacardium* and *Mangifera* emit a strong and sweet scent suggesting the possible presence of osmophores. Surprisingly, we realized that scent glands were never mentioned in the family. Our study confirms the presence of osmophores in both genera, which are structurally similar. However, the composition of the fragrance shows varies widely between species, with sesquiterpenes dominating the scents of *Anacardium humile* and monoterpenes in *Mangifera indica*. Slight variations in their subcellular apparatus are consistent with those found in the composition of their fragrance.

## Introduction

Pleasant or not, the fragrance of a flower typically serves as a sensory cue for pollinators, often indicative of some kind of reward, notably nectar (Vogel 1990; Endress 1994). Like nectar, floral scents are produced by specialized structures commonly referred to as scent glands or osmophores, which are located mainly on petals and other floral organs, such as sepals or stamens, or other specialized reproductive structures (Vogel 1990; Sazima *et al.* 1993; Vogel and Hadacek 2004; Guimarães *et al.* 2008; Melo *et al.* 2010; Marinho *et al.* 2014; Possobon *et al.* 2015; Gagliardi *et al.* 2016).

Osmophores typically consist of an epidermis of specialized secretory cells and/or secretory parenchyma. They are concentrated in certain regions of the floral organs and can have different shapes, sizes and colours (Vogel 1990; Vogel and Hadacek 2004; Guimarães *et al.* 2008; Melo *et al.* 2010; Marinho *et al.* 2014; Possobon *et al.* 2015; Gagliardi *et al.* 2016; Gonçalves-Souza *et al.* 2017). Osmophores are found in many unrelated orders of flowering plants, occurring on the distal portion of the spadix of some Araceae (Alismatales) and petals of Iridaceae or Orchidaceae (Asparagales) in monocots, on petals of Euphorbiaceae and the corona of some Passifloraceae (Malpighiales), on petals of Fabaceae (Fabales) as well as the anthers of Solanaceae (Solanales) or the staminal appendages (‘horns’) of Asclepiodoideae (Gentianales), among other eudicots (Vogel 1990; Evert 2006; Teixeira *et al.* 2014; Marinho *et al.* 2014, 2018; Gagliardi *et al.* 2016; Demarco 2017a).

Flowers of most members of Sapindales are nectariferous and often have a conspicuous nectary (Kubitzki 2011; Ronse De Craene and Haston 2006). However, out of the nine families and ca. 6550 species (Stevens 2001 onwards), osmophores have only been reported and studied in two genera of Rutaceae (Bussel *et al.* 1995; Marques *et al.* 2015), while those reported in two species of a genus of Sapindaceae were not studied in detail and lacked chemical characterization of their volatile compounds (Lima *et al.* 2016).

Anacardiaceae are one of the largest families of Sapindales and comprise 82 genera and ~800 species distributed mainly in tropical areas (Pell *et al.* 2011; Weeks *et al.* 2014). As in all other members of the order, the flowers of most genera of the family show features typical of melittophily, such as diurnal anthesis, small dish-shaped flowers and a conspicuous nectary often in the shape of a fleshy intrastaminal disk (Pell *et al.* 2011). During previous studies we noticed that, aside from producing nectar, the flowers of *Anacardium humile*, *A. occidentale* and *Mangifera indica* also emitted a strong and sweet scent suggesting the possible presence of osmophores (Bachelier and Endress 2009; Tolke *et al.* 2015,^2018^). Surprisingly, we realized that such secretory structures were never mentioned, not even in *A. occidentale* and *M. indica*, which are two economically important and extensively studied fruit crop species (Mukherjee 1972; Johnson 1973; Schnell *et al.* 1995; Freire *et al.* 2002).

In this study, we localized and characterized previously unrecognized specialized epidermal cells located at the base of the adaxial side of each petal of *A. humile* and *M. indica* and based on their secretory function, interpret them here as osmophores. Their ultrastructural features were studied before and during anthesis in order to understand their mechanism of secretion and whether there is a link between their subcellular apparatus and the composition of the floral fragrance by examining flowers of each species by gas chromatography–mass spectrometry (GC-MS). Based on our results, we also discuss the importance of diversified strategies on the attraction of pollinators in Anacardiaceae and the entire Sapindales, despite the scarcity of reports of osmophores in the order.

## Materials and Methods

### Plant material

Floral buds and flowers of *A. humile* were collected in the ‘Reserva Biológica e Estação Experimental de Mogi-Guaçu’, state of São Paulo, Brazil. Floral buds and flowers of *M. indica* were collected from plants cultivated at the gardens of the ‘Universidade Estadual de Campinas’, state of São Paulo, Brazil. Voucher specimens were deposited in the herbarium UEC (*A. humile*, UEC 119573; *M. indica*, UEC 119571).

### Location and structure of the osmophores

All collected material was fixed in BNF (buffered neutral formalin) for 48 h (Lillie 1965) and stored in 70 % ethanol. The material was dehydrated through a tertiary butyl alcohol series and embedded in Paraplast® (Merck KGaA, Darmstadt, Germany) (Johansen 1940). Transverse and longitudinal sections 8–10 µm thick were obtained using a Leica RM2245 rotary microtome (Leica Microsystems Richmond, Inc., Wetzlar, Germany) and stained with Astra blue (Merck KGaA) and safranin (C.I. 50240, Merck KGaA) (Gerlach 1984). In order to detect the presence of lipids, some sections were stained with Sudan black B (C.I. 26150; Pearse 1985). All slides were mounted in Entellan® synthetic resin (Merck KGaA), and the images were obtained with an Olympus DP71 digital camera coupled to an Olympus BX51 microscope.

The anthetic flowers were dissected under a Leica M80 stereomicroscope (Leica Biosystems Richmond, Inc., Wetzlar, Germany) and dehydrated in an ethanol dilution series before being critically point-dried with CO_2_ and sputter-coated with gold (SCD-050 sputter coater, Bal-Tec AG, Balzers, Liechtenstein). Observations were carried out using a JEOL JSM 5800 LV scanning electron microscope (JEOL, Tokyo, Japan).

### Ultrastructural organization of the osmophores

Petals of flowers in pre-anthesis and anthesis were fixed in 2.5 % glutaraldehyde in 0.1 M phosphate buffer, pH 7.3 for 24 h at 4 °C. They were post-fixed in 1 % osmium tetroxide in the same buffer for 1 h at room temperature, dehydrated in an acetone dilution series and embedded in Araldite resin (Machado and Rodrigues 2004). Ultrathin sections were obtained with a diamond knife and counterstained with uranyl acetate (Merck KGaA) (Watson 1958) and lead citrate (Merck KGaA) (Reynolds 1963). The material was observed with a Tecnai G2 Spirit Bio TWIN transmission electron microscope (FEI Company, Hillsboro, OR, USA).

### Floral bouquet composition

Entire fresh flowers of *A. humile* (27.49 g) and *M. indica* (16.2 g) were submitted (separately) to hydrodistillation in a Clevenger-type apparatus for 4 h. The crude oils were extracted with dichloromethane, dried (anhydrous) Na_2_SO_4_, filtered, and the solvent removed at room temperature. The oils obtained were kept at –20 °C in amber glass bottles until the identification of their chemical composition. The extraction yield of each essential oil was expressed in % (w/w) of the fresh flowers.

Gas chromatography–mass spectrometry analysis of the volatile oils was performed with an Agilent 6850 gas chromatograph (Palo Alto, CA, USA) equipped with a HP-5MS capillary column (30 m × 0.25 mm i.d.; 0.25 μm) and connected to an Agilent 5975C mass spectrometer operating in the positive ion electron impact ionization (70 eV; m/z 50–800; source temperature 280 °C; quadruple temperature 180 °C). The column temperature was initially maintained at 50 °C for 5 min, increased to 100 °C at 3 °C min^−1^, and subsequently increased to 325 °C at 10 °C min^−1^, and maintained for 7 min at 325 °C. The carrier gas was helium at a flow rate of 1.0 mL min^−1^. The inlet temperature was maintained at 320 °C at a split mode of 50:1. The compounds were identified from their retention indices (Adams 2001) and by interpreting their fragmentation patterns in the mass spectra, further confirmed by comparing with fragmentation patterns of authentic compounds and the relevant spectral data from the NIST and Wiley libraries.

## Results

### Location and structure of the osmophores

**Figure 1.**
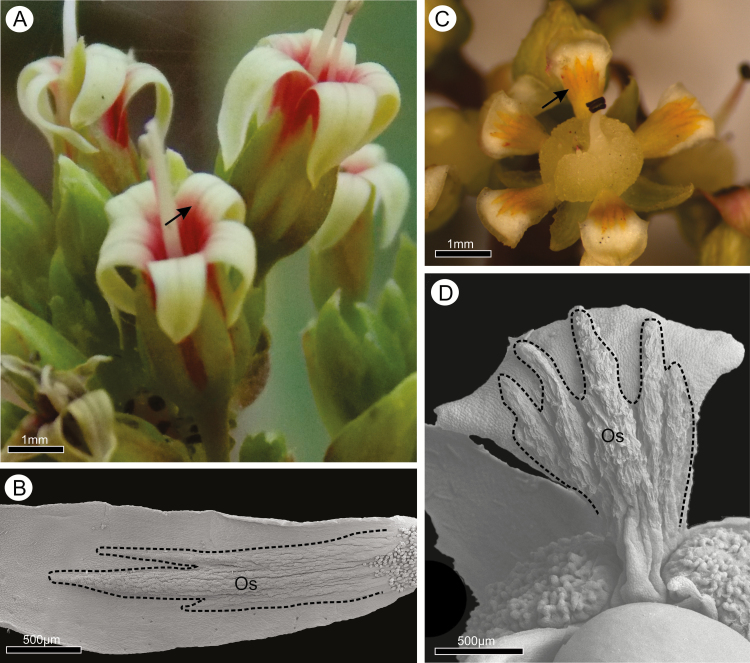
Flowers of *Anacardium humile* (A and B) and *Mangifera indica* (C and D) in fresh material and scanning electron microscope images showing osmophores on the ridges of the adaxial surface (arrow) of petals. Os, osmophore.

In both species, the adaxial surface at the base of each petal displayed prominent longitudinal ridges covered with distinct epidermal cells that were here interpreted as osmophores (Fig. 1). In *A. humile*, the osmophores were found to be located on narrow ridges following the main vascular bundles from the base up to the longitudinal midpoint of the petals. Their colour was recorded as white in anthetic flowers like the other regions of the petals, but turning distinctly pink later (Fig. 1A and B). In *M. indica*, the osmophores were found to be located not only on the ridges but also covering most of the lower half of the petals. Their colour was yellow at anthesis, turning brown later (Fig. 1C and D).

**Figure 2.**
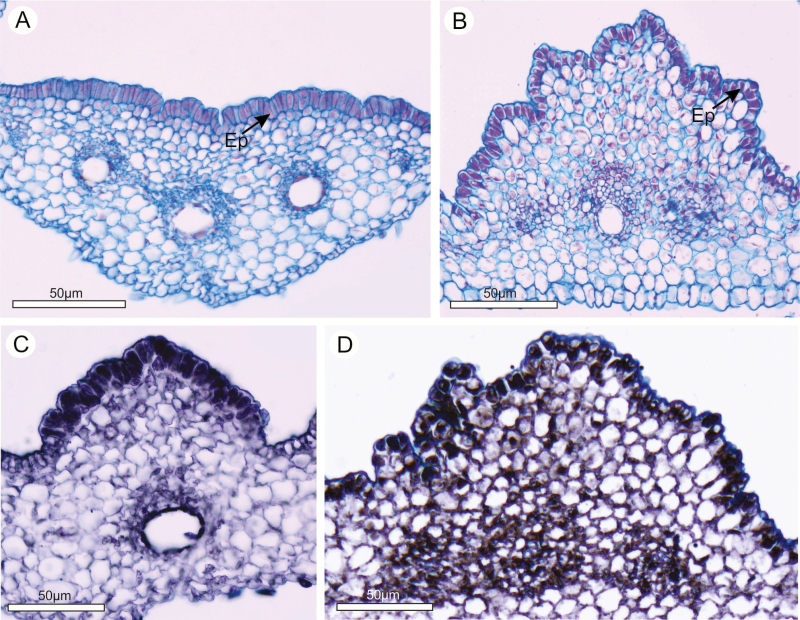
Anatomy (A and B) and histochemical tests (C and D) in cross-sections. (A) Palisade-like osmophore of *Anacardium humile*. (B) Osmophore of *Mangifera indica* formed by epidermal cells; note that the shape is like the underlying cells. (C) Positive reaction to Sudan black B on the epidermal cells of the petals of *A. humile*. (D) Positive reaction to Sudan black B on the epidermal cells of the petals of *M. indica*. Ep, epidermis.

The osmophores were composed of only one layer of epidermal cells characterized by a prominent nucleus and a densely stained cytoplasm (Fig. 2A and B). A positive reaction for lipids confirmed the oil content in both species (Fig. 2C and D). In *A. humile*, the epidermis appeared as distinctly palisadic cells (Fig. 2A), whereas in *M. indica* their shapes were more irregular, ranging from cubic to elongated, like those of the mesophyll beneath (Fig. 2B). There was no evidence of stomata or vascular supply directed towards the osmophore.

### Ultrastructural organization of the osmophores

**Figure 3.**
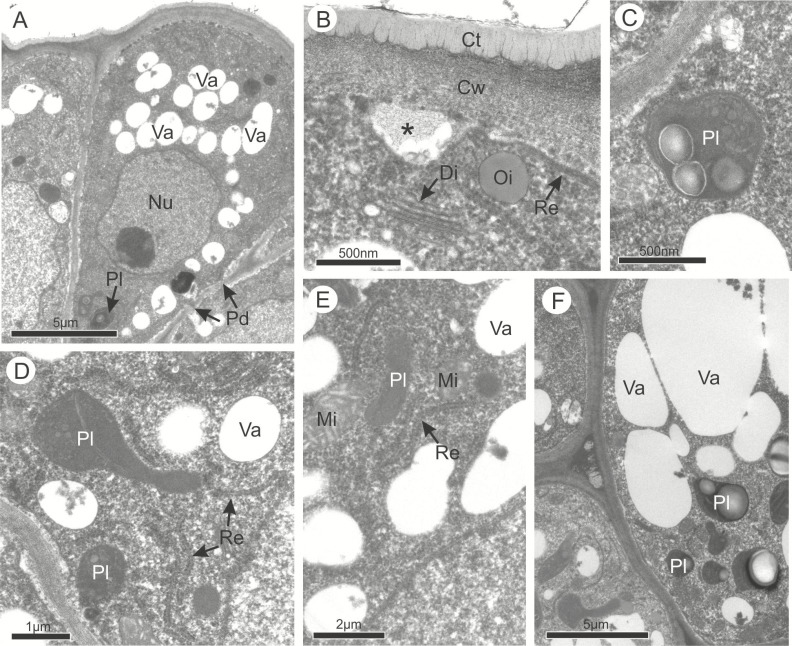
Ultrastructural features of the osmophores in *Anacardium humile* in pre-anthesis. (A) General view of the epidermal cells. (B) Cuticle. Note the presence of vacuoles, oil droplets and dictyosomes near to the plasma membrane. The secretion is observed in the periplasmic space (*). (C) Plastid containing starch. (D and E) Epidermal cell showing polyribosomes, mitochondria, vacuoles, plastids and rough endoplasmic reticulum. (F) Large vacuoles and plastids containing starch. Ct, cuticle; Cw, cell wall; Di, dictyosomes; Mi, mitochondria; Nu, nucleus; Oi, oil droplet; Pd, plasmodesma; Pl, plastid; Re, rough endoplasmic reticulum; Va, vacuole.

**Figure 4.**
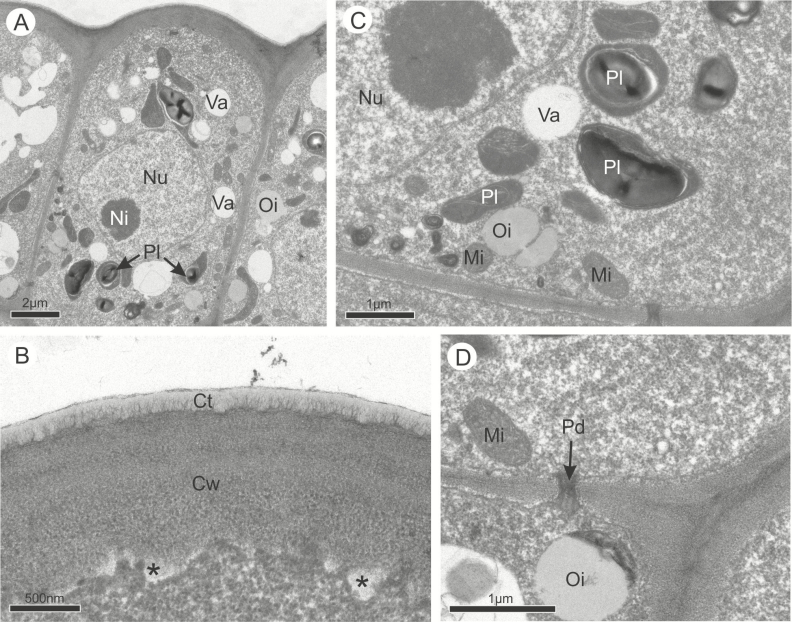
Ultrastructural features of the osmophores in *Mangifera indica* in pre-anthesis. (A) General view of the epidermal cells. (B) Cuticle. Asterisks indicate the secretions stored between the plasma membrane and the cell wall. (C) Epidermal cell with polyribosomes, mitochondria, plastids containing starch, vacuoles and oil droplets. (D) Plasmodesma between epidermal cells. Ct, cuticle; Cw, cell wall; Mi, mitochondria; Ni, nucleolus; Nu, nucleus; Oi, oil droplet; Pd, plasmodesma; Pl, plastid; Re, rough endoplasmic reticulum; Va, vacuole.

Despite their differences in shape and size, the cells of the osmophores in both species had the same subcellular bipolar organization and characteristics that indicate their similar secretory function (Figs 3A–F and 4A–F). Before anthesis, TEM observations revealed that in both species the epidermal cells have a thicker outer periclinal wall covered by a thin cuticle (Figs 3B and 4B), and dense and organelle-rich cytoplasm interconnected via plasmodesmata in the anticlinal walls (Figs 3A, D, E and 4A, C, D). In the proximal region, where the fragrant compounds are synthetized, all the cells had a large nucleus surrounded by a rough endoplasmic reticulum, and a high number of polyribosomes, mitochondria, small vacuoles and plastids containing starch grains and numerous oil droplets free in the cytosol (Figs 3A–F and 4A–F). In both species, some of the cells contained larger vacuoles (Fig. 3E and F) and a larger population of plastids containing starch grains (Figs 3F and 4A, C). In the apical portion where the secretions are released, the cytoplasm appeared as less dense than in the proximal region due to a greater number of vesicles and vacuoles of various sizes (Figs 3A and 4A). In *A. humile*, dictyosomes occurred in larger quantity with more vesicles than in *M. indica* (Fig. 3B), and the oil droplets were more common, always located in a peripheral position near the dictyosomes and near the endoplasmic reticulum (Figs 3B and 4C). In both species, secretion was observed in the periplasmic space and released by vesicles and vacuoles which merge with the plasma membrane on the apical side of the cell (Figs 3B and 4B). The cuticle displayed pectin projections in both species, being slightly thicker in *A. humile* than in *M. indica* (Figs 3B and 4B).

**Figure 5.**
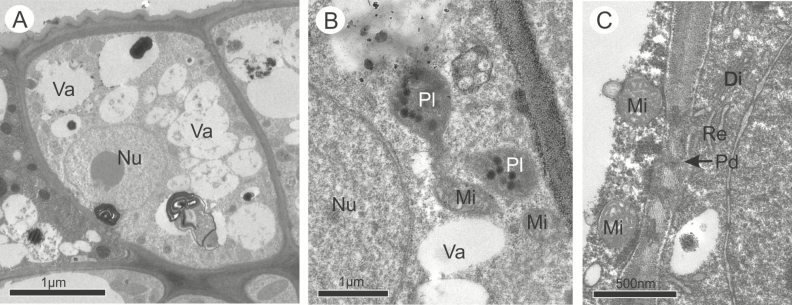
Ultrastructural features of the osmophores in *Anacardium humile* at anthesis. (A) General view of the osmophores. (B) Epidermal cell showing the plastids containing oil droplets. (C) Plasmodesmata. Note also the presence of mitochondria, dictyosomes and endoplasmic reticulum. Di, dictyosomes; Mi, mitochondria; Nu, nucleus; Pd, plasmodesma; Pl, plastid; Re, endoplasmic reticulum; Va, vacuole.

**Figure 6.**
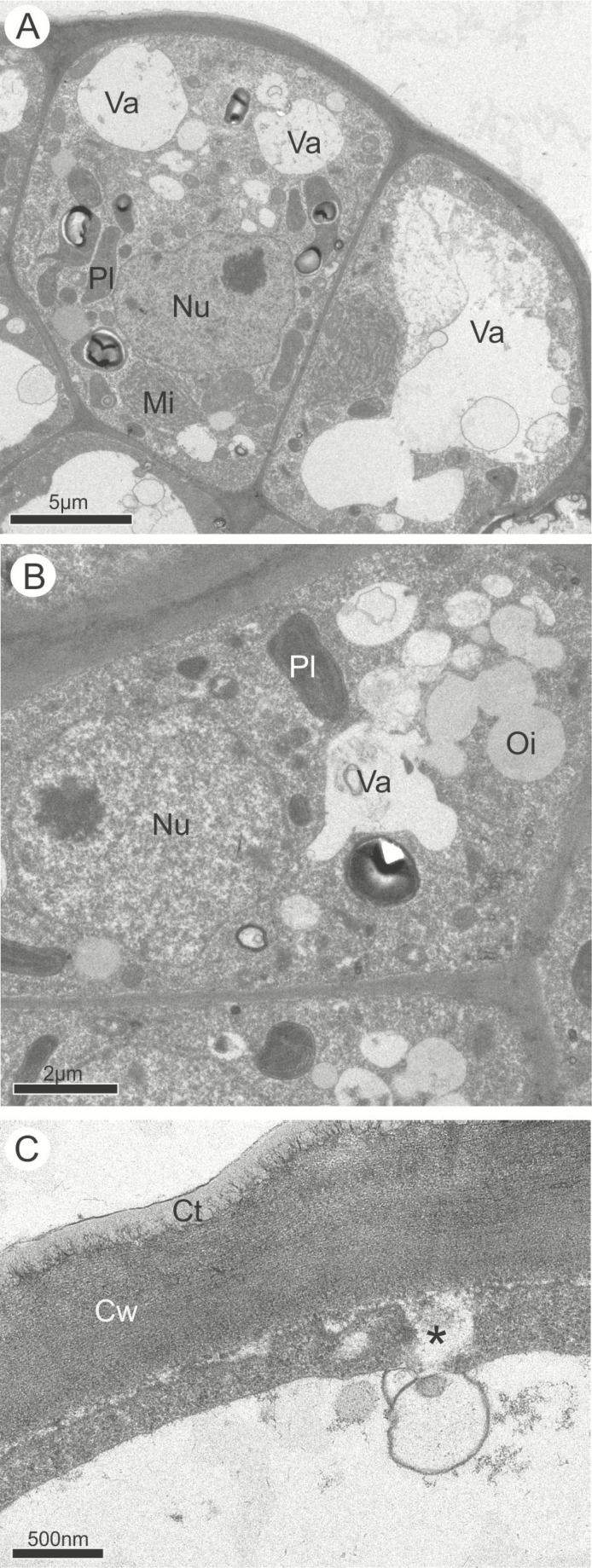
Ultrastructural features of the osmophores in *Mangifera indica* at anthesis. (A) General view of the osmophore during anthesis. Most of the cells contain a single large vacuole occupying almost the entire cytoplasm and the organelles are in a peripheral position; the cells are in different stages of secretion. (B) Small vacuoles and oil droplets dispersed in the cytoplasm of the epidermal cells. (C) Fusion of the vacuole with the cell wall before the release of the secretion (*). Ct, cuticle; Cw, cell wall; Mi, mitochondria; Nu, nucleus; Oi, oil droplet; Pl, plastid; Va, vacuole.

During anthesis, the vacuoles were larger than during the previous phase and in both species some cells were entirely occupied by a single and large vacuole filled with membranous and flocculent material while others were at different stages of secretion (Figs 5A–C and 6A–C). There was no difference in types of organelles found in the previous phase, and only their numbers were quite reduced (Figs 5A–C and 6A–C). In the distal portion of the active cells, vesicles and small vacuoles continued to merge with the plasma membrane and released their secretions to the periplasmic space which crossed the outer periclinal cell wall and the cuticle without disruption (Fig. 6C). Only in *A. humile* there was a total depletion of starch with a concomitant emergence of plastoglobules within plastids (Fig. 5B).

### Floral bouquet composition

**Figure 7.**
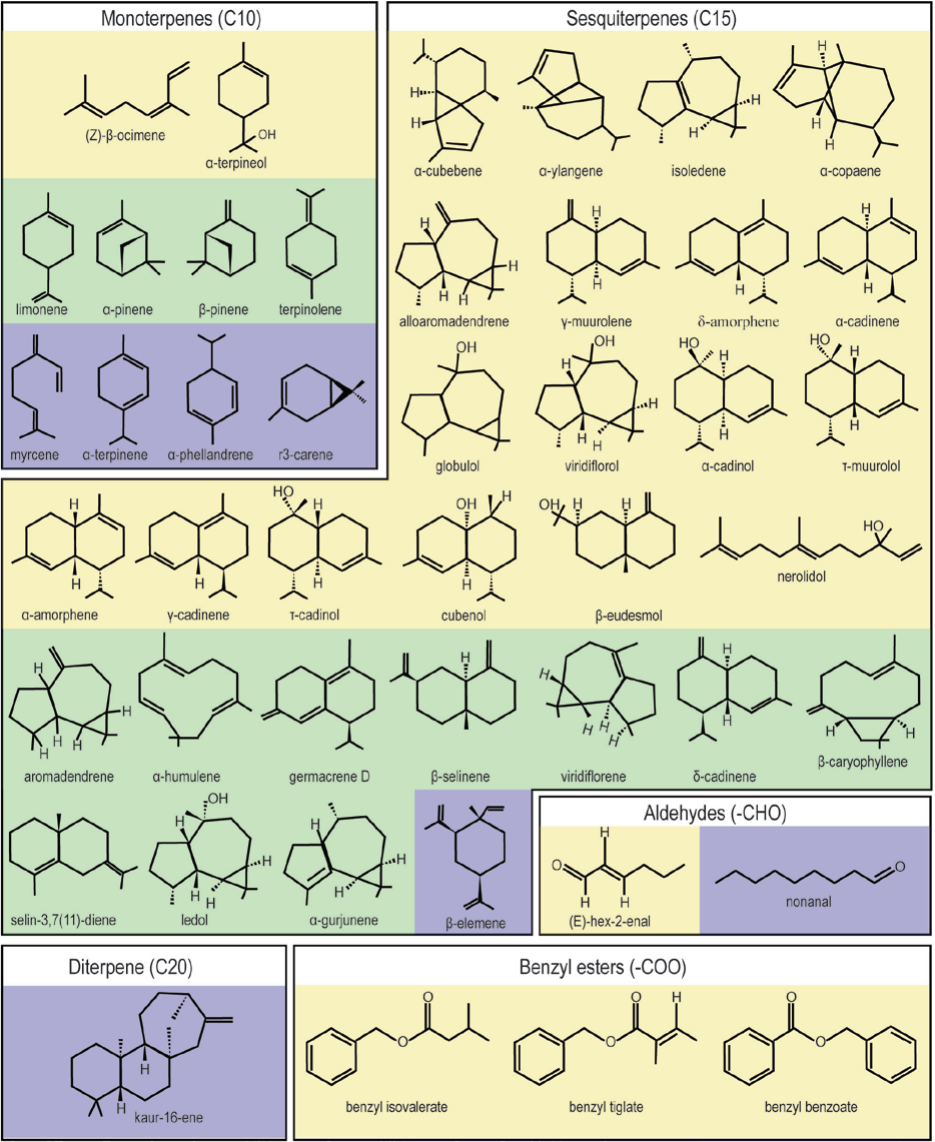
Diversity of volatile compounds produced by the flowers of *Anacardium humile* (24 in yellow) and *Mangifera indica* (7 in blue). The compounds produced by both species are represented in green (14).

Our GC-MS analyses revealed that out of the 39 volatile compounds produced by the flowers in *A. humile* and 21 in *M. indica*, 14 are identical (10 sesquiterpenes and 4 monoterpenes). Diverse sesquiterpenes and monoterpenes occurred in both species and these dominate the composition of the fragrance in both cases (Fig. 7). They represent a total of 84.03 % of the compounds identified in *A. humile* and 96.78 % in *M. indica*. However, their relative concentrations differed markedly (e.g. the sesquiterpene β-caryophyllene or the monoterpene α-pinene) as well as their general presence/absence in each species (e.g. the sesquiterpene isoledene and the monoterpene (Z)-β-ocimene in *A. humile* and the sesquiterpene elemene and the monoterpene myrcene in *M. indica*) (Table 1). The sesquiterpenes represent 73.24 % of the fragrance in *A. humile* and are dominated by β-caryophyllene (29.36 %), followed by α-humulene (11.76 %) and δ-cadinene (7.05 %). In contrast, *M. indica* produced mainly monoterpenes (75.64 %), dominated by terpinolene (55.95 %), α-pinene (5.84 %) and Δ^3^-carene (5.12 %) (Table 1). In addition to sesqui- and monoterpenes, benzyl esters and oxygenated monoterpenes were also detected but only in *A. humile*, and diterpenes only in *M. indica* (Table 1; Fig. 7).

**Table 1.** Relative yields (%) of the volatile oils from flowers of *Anacardium humile* and *Mangifera indica* organized by chemical classes and RI. ^a^RI: retention indices relative to C_6_–C_30_*n*-alkanes on the same column; components identified through RI and MS.

Component	Chemical class	RI^a^	*A. humile*	*M. indica*
(E)-Hex-2-enal	Aldehyde	855	1.36	–
α-Pinene	MonHy	938	8.89	5.84
β-Pinene	MonHy	979	0.43	0.77
Myrcene	MonHy	990	–	3.30
Δ^3^-Carene	MonHy	1011	–	5.12
α-Terpinene	MonHy	1017	–	0.44
α-Phellandrene	MonHy	1030	–	0.79
Limonene	MonHy	1031	0.76	3.43
(Z)-β-Ocimene	MonHy	1040	0.41	–
Terpinolene	MonHy	1087	0.30	55.95
Nonanal	Aldehyde	1102	–	0.40
α-Terpineol	OxyMo	1188	0.24	–
α-Cubebene	SesHy	1351	0.82	–
α-Ylangene	SesHy	1372	0.31	–
Isoledene	SesHy	1373	0.38	–
α-Copaene	SesHy	1375	3.49	–
Benzyl isovalerate	BenEs	1382	0.54	–
β-Elemene	SesHy	1391	–	0.35
α-Gurjunene	SesHy	1408	0.48	6.00
β-Caryophyllene	SesHy	1418	29.36	3.66
Aromadendrene	SesHy	1439	1.14	0.49
α-Humulene	SesHy	1454	11.76	2.04
Alloaromadendrene	SesHy	1461	2.47	–
γ-Muurolene	SesHy	1477	2.84	–
Germacrene D	SesHy	1480	0.37	0.14
β-Selinene	SesHy	1485	0.97	5.96
Viridiflorene	SesHy	1493	5.51	2.10
Benzyl tiglate	BenEs	1496	4.07	–
δ-Amorphene	SesHy	1497	0.69	–
α-Muurolene	SesHy	1499	0.85	–
α-Amorphene	SesHy	1506	1.43	–
γ-Cadinene	SesHy	1513	1.86	–
δ-Cadinene	SesHy	1524	7.05	0.19
α-Cadinene	SesHy	1538	1.11	–
Selin-3,7(11)-diene	SesHy	1542	0.35	0.21
Nerolidol	OxySe	1564	1.31	–
Ledol	OxySe	1565	0.20	0.27
Globulol	OxySe	1583	1.42	–
Viridiflorol	OxySe	1590	0.81	–
τ-Cadinol	OxySe	1640	1.14	–
τ-Muurolol	OxySe	1641	1.31	–
Cubenol	OxySe	1642	0.45	–
β-Eudesmol	OxySe	1649	0.33	–
α-Cadinol	OxySe	1653	0.44	–
Benzyl benzoate	BenEs	1762	1.93	–
Kaur-16-ene	Diterpene	2034	–	0.23
**Total identified**			99.58	97.68
Monoterpenes^MonHy^			10.79	75.64
Oxygenated monoterpenes^OxyMo^			0.24	–
Sesquiterpenes^SesHy^			73.24	21.14
Oxygenated sesquiterpenes^OxySe^			7.41	0.27
Others (including aldehydes, benzyl esters^BenEs^ and diterpenes)			7.90	0.63
**Volatile oil yield [% (w/w)]**			0.0051 %	0.0093 %

## Discussion

### Diversity of osmophores in flowers of Anacardiaceae and other Sapindales

In the present study, we describe the presence of specialized secretory epidermal cells concentrated at the base of the petals of *A. humile* and *M. indica*—interpreted here as osmophores (see below subcellular apparatus). Coincidentally, both species were previously placed in Mangiferae (*sensu*Engler 1892) and now, together with most other former members of this tribe, form a well-supported monophyletic clade nested in Anacardioideae, the largest of the two subfamilies of Anacardiaceae (Weeks *et al.* 2014). In both species, the osmophores are conspicuously situated along ridges of the adaxial (ventral) side of petals; a similar structure is also sometimes visible in pictures and sections of other members of the family, such as *Pleiogynium solandri* in Spondioideae, and *Semecarpus riparius* and *S. australiensis* in Anacardioideae (Wannan and Quinn 1991; Bachelier and Endress 2009). However, a careful re-examination of the sections of these genera and those studied by Bachelier and Endress (2009; see list of material therein) confirmed the secretory activity of the epidermal cells on these ridges only in *Anacardium occidentale* and *M. indica*, and its absence in all other taxa. Only one previous study using neutral red suggested that osmophores may also be present on the adaxial side and tips of the petals of *Tapirira guianensis* (Fernandes *et al.* 2012), which belong to the subfamily Spondioideae. However, they did not report any scent, and staining with neutral red is not always conclusive (Gonçalves-Souza *et al.* 2017; Demarco 2017b). Our study is thus the first to provide anatomical evidence of the production of volatiles in Anacardiaceae, and more studies are necessary to confirm the presence of scented flowers and potential osmophores in more members of the family.

In other families of the Sapindales, the presence of osmophores was reported only in a few other species. In Rutaceae, osmophores have been described in a few species of *Citrus* on the tips of the petals and consist of a distinctly papillose epidermis with glandular trichomes (Marques *et al.* 2015). Scents have also been identified in flowers of *Boronia*; however, the authors could not confirm whether osmophores were responsible for its production (Bussel *et al.* 1995). In Sapindaceae, osmophores have been reported in *Paullinia weinmanniifolia* on the stamens and sepals (Lima *et al.* 2016), but neither with information on their structure, nor, like in *Citrus*, with details on their ultrastructure. Therefore, comparisons of osmophore diversity and evolution in Sapindales are still hampered by the lack of detailed studies.

### Synthesis and release of volatile compounds produced by the osmophores

The secretory nature of the osmophores of *A. humile* and *M. indica* was here clearly demonstrated by the presence of cells with high metabolic activity. Their subcellular apparatus is essentially similar to that typically described in osmophores of other flowering plants (Fahn 1979, 2000; Davies *et al.* 2003; Ascensão *et al.* 2005; Antón *et al.* 2012; Pansarin *et al.* 2014). In the epidermis, the accumulation of starch grains observed here in both species is a very common feature of osmophores (Stern *et al.* 1987; Pansarin *et al.* 2009; Melo *et al.* 2010). These carbohydrates provide the main source of energy for the mitochondria and for the production of the volatiles (Fahn 1979; Vogel 1990; Melo *et al.* 2010; Kowalkowska *et al.* 2015; Gonçalves-Souza *et al.* 2017). Smooth endoplasmic reticulum, leucoplasts, polyribosomes and dictyosomes are also very common in cells that, like those of both species, produce terpenes (Turner and Croteau 2004; Melo *et al.* 2010; Marinho *et al.* 2018). According to these studies, plastids and rough endoplasmic reticulum are the main organelles responsible for the production of terpenes, and small vacuoles or plastids with plastoglobuli are indicative of their production. In addition, the presence of cytoplasmic oil droplets in both species confirms the production of oils (Effmert *et al.* 2006; Fahn 1979, 2000; Turner and Croteau 2004; Melo *et al.* 2010; Gonçalves-Souza *et al.* 2017; Marinho *et al.* 2018).

There are some slight variations in the subcellular apparatus of each species studied here, which seem to be consistent with the variation of the composition of the floral fragrance. Sesquiterpenes, such as those dominant in *A. humile*, are produced in the cytosol, whereas monoterpenes and diterpenes, such as those found more often in *M. indica*, are mainly produced by the plastids which are more numerous in this species (Aharoni *et al.* 2005; Effmert *et al.* 2006). However, the isoprenoid precursors found in both genera may be available in different cell compartments and may be used to generate terpenoids in more than one cell compartment (Aharoni *et al.* 2005; Effmert *et al.* 2006). In addition, benzyl esters are primarily synthesized in the plastids through the arogenate pathway and modified by the enzymes present in the cytosol (Maeda and Dudareva 2012), and the fatty acid derivatives, such as aldehydes, alcohols and their esters are produced by both plastids and peroxisomes (Borghi *et al.* 2017). Only plastoglobules were found exclusively in *A. humile* and may thus play a role in the dominant production of the sesquiterpenes (Pacek *et al.* 2012; Gonçalves-Souza *et al.* 2017).

Our study also demonstrates that in both species, all secretions, including highly lipophilic organic compounds such as terpenoids, are released by a granulocrine mechanism across the plasma membrane and into the periplasmic space via active transport, and then cross the cell wall and cuticle as it is usually observed in the release of lipophilic compounds (Curry *et al.* 1988; Fahn 2000; Riederer 2006; Pansarin *et al.* 2014; Gama *et al.* 2015; Kowalkowska *et al.* 2015; Possobon *et al.* 2015; Paiva 2016; Gonçalves-Souza *et al.* 2017). Ultrastructural studies like this one have not been conducted before in Sapindales. The granulocrine mechanism has been described for osmophores of other unrelated families of eudicots, such as Fabaceae and Passifloraceae, or for monocots such as Araceae and Orchidaceae (Pridgeon and Stern 1983; Skubatz *et al.* 1995; García *et al.* 2007; Gonçalves-Souza *et al.* 2017; Marinho *et al.* 2018). Moreover, the extensive production and release of scents is suggested by the presence of cells at different stages of secretion in anthetic flowers (Fahn 1979, 2000), an important strategy to attract pollinators over a longer period of time.

### Diversity of volatile compounds in the floral bouquet of Anacardiaceae

Our study demonstrates that the flowers of both species produce sesquiterpenes and monoterpenes as primary volatile components of their fragrance, like most of the angiosperms that contain osmophores (Metcalf and Kogan 1987; Knudsen *et al.* 2006). The fragrance is dominated by sesquiterpenes in *A. humile*, especially β-caryophyllene, and, as previously reported by Wang *et al.* (2010), monoterpenes, especially terpinolene, in *M. indica*. The same types of substances are also identified as main components of essential oils in fruits and leaves of both species. However, in these organs, the predominant sesquiterpenes in *A. humile* are α-bulnesene and γ-cadinene (Cardoso *et al.* 2010; Winck *et al.* 2010), and in leaves of *M. indica* the principal monoterpene is cyperene (Gebara *et al.* 2011).

Some of the sesquiterpenes present in both species have been shown to be insect kairomones, which are efficient attractants of Hymenoptera (γ-cadinene) and Coleoptera (β-caryophyllene, α-copaene, α-pinene, among others) (Metcalf and Kogan 1987; Knudsen *et al.* 2006). This supports previous studies demonstrating that the main pollinators in both species are honeybees and beetles, as well as flies, which are more likely attracted by these compounds (Free and Williams 1976; Anderson *et al.* 1982; Jirón and Hedstrom 1985; Mitchell and Mori 1987; Heard *et al.* 1990; Freitas and Paxton 1996; Senchina and Summerville 2007; Almeida *et al.* 2011). Such diversity of pollinators and subtle differences in the sets of compounds comprised in the floral scents of both species likely reflects different functions and additional levels of specialization regarding pollinator behaviour and attraction (Azuma *et al.* 1997; Anderson *et al.* 2002). For instance, some components, such as benzenoids and terpenoids, are known to have stronger effects over long distances than other volatiles, that often only excerpt their action in small local ranges in order to induce feeding or to indicate the presence of floral rewards, especially nectar (e.g. aldehydes) (Azuma *et al.* 1997; Dötterl and Jürgens 2005; Knudsen *et al.* 2006; Raguso 2008; Wright and Schiestl 2009; Junker and Blüthgen 2010).

Further studies should focus on compounds produced by isolated parts of flowers in order to determine whether other floral secretory structures, aside from osmophores, also participate in the synthesis of scents. Gonçalves-Souza *et al.* (2017) demonstrated that different floral organs of Araceae may produce scents in a same flower. However, in Anacardiaceae there seems to be a clear contribution to the overall scent by secretions produced by the ducts, complicating the possible analysis of scents produced by individual separated floral organs.

### Scent production in nectariferous flowers of Anacardiaceae and other Sapindales

The floral scents are an important way of communication between the flowers and their pollinators. They indicate the presence of a reward offered by the flowers, most of the time nectar (Vogel 1990; Endress 1994; Raguso 2008). The association between nectar and fragrance production can be quickly learned by social bees (Wright 2009), which are major pollinators of Anacardiaceae and Sapindales in general (Kubitzki 2011). In all families of the Sapindales for which the presence of osmophores has been reported, nectaries are also present as in the two species studied here (Bussel *et al.* 1995, Marques *et al.* 2015; Lima *et al.* 2016). Indeed, most members of the order share generalist to melittophilous insect pollination syndromes, with numerous but relatively small nectariferous flowers with a polysymmetric perianth (and androecium) (Engler 1892; Wannan and Quinn 1991; Bachelier and Endress 2009; Kubitzki 2011; Tölke *et al.* 2015). Since most other members of the order produce nectar, it is surprising that there are to date only a few reports of scented flowers mainly in Meliaceae, Rutaceae and Sapindaceae (Kubitzki 2011). In addition, most members of Sapindales produced (terminal or distal) inflorescences with numerous flowers which sometimes have a conspicuous fleshy nectary disc, but individual flowers are often small and their perianth inconspicuous, and appear, at least to the human eye, to not present any other obvious visual cues that indicate a reward (Endress 2010; Kubitzki 2011). Therefore, scented flowers and osmophores in flowers of Anacardiaceae and other sapindalean families have either been overlooked until now, or rely on other strategies to attract pollinators, which can only become evident with further studies.

## Conclusion and Perspectives

This is the first study confirming the presence of osmophores in members of Anacardiaceae and providing details on their structure and function. It also reveals that while in both species the presence of a reward is advertised with a strong fragrance, still little is known on the evolution of scent and other cues signalling the presence of a reward present in most other members of Anacardiaceae and Sapindales. The results encourage further investigations on the possible presence of fragrances in other genera of the family and order, into the origin and composition of such fragrances and the relationship between their diversity and their pollination mechanisms.

## Sources of Funding

This research was financially supported by grants from ‘Fundação de Amparo à Pesquisa do Estado de São Paulo’ (FAPESP - proc. nº 2014/18002-2) and CNPq (proc. nº 420417/2016-8). The first author also thanks CAPES for a scholarship provided for a semester abroad at FU Berlin (PDSE proc. nº 88881.133676/2016-01).

## Contributions by the Authors

E.D.T. and D.D. posed the central questions; E.A.L. carried out the field work; E.D.T., D.D. and S.M.C. performed the microscopic analysis; M.J.P.F. performed the chemistry analysis; the authors analysed the data together; E.D.T. wrote the original manuscript and D.D. and J.B.B. edited for content and provided guidance on structure and style.

## Conflict of Interest

None declared.
